# The Role of Costimulation Blockade in Solid Organ and Islet Xenotransplantation

**DOI:** 10.1155/2017/8415205

**Published:** 2017-10-11

**Authors:** Kannan P. Samy, James R. Butler, Ping Li, David K. C. Cooper, Burcin Ekser

**Affiliations:** ^1^Division of Transplant Surgery, Department of Surgery, Indiana University School of Medicine, Indianapolis, IN, USA; ^2^Xenotransplantation Program, Department of Surgery, The University of Alabama at Birmingham, Birmingham, AL, USA

## Abstract

Pig-to-human xenotransplantation offers a potential bridge to the growing disparity between patients with end-stage organ failure and graft availability. Early studies attempting to overcome cross-species barriers demonstrated robust humoral immune responses to discordant xenoantigens. Recent advances have led to highly efficient and targeted genomic editing, drastically altering the playing field towards rapid production of less immunogenic porcine tissues and even the discussion of human xenotransplantation trials. However, as these humoral immune barriers to cross-species transplantation are overcome with advanced transgenics, cellular immunity to these novel xenografts remains an outstanding issue. Therefore, understanding and optimizing immunomodulation will be paramount for successful clinical xenotransplantation. Costimulation blockade agents have been introduced in xenotransplantation research in 2000 with anti-CD154mAb. Most recently, prolonged survival has been achieved in solid organ (kidney xenograft survival > 400 days with anti-CD154mAb, heart xenograft survival > 900 days, and liver xenograft survival 29 days with anti-CD40mAb) and islet xenotransplantation (>600 days with anti-CD154mAb) with the use of these potent experimental agents. As the development of novel genetic modifications and costimulation blocking agents converges, we review their impact thus far on preclinical xenotransplantation and the potential for future application.

## 1. Introduction

Organ transplantation remains the definitive treatment for patients suffering from end-stage organ failure. Unfortunately, this treatment remains severely limited due to the critical shortage of suitable allografts for transplantation [1, 2]. The use of genetically engineered pigs as a supplemental source of tissues or organs offers a promising answer to this dilemma [3]. Pig-to-human xenotransplantation has been pursued for more than a century; however, early studies demonstrated substantial barriers to clinical application in the form of hyperacute rejection, acute humoral xenograft rejection (AHXR), and thrombosis [4, 5].

The modern era of xenotransplantation was stimulated by the identification of the Gal *α*(1,3) Gal (Gal) porcine epitope and its role in early rejection [6–8]. The subsequent advent of *α*1,3-galactosyltransferase gene knockout (GTKO) pigs eliminated a major barrier to xenotransplantation by negating the role of high percentage of human xenoreactive antibodies [9, 10]. However, residual preformed human antibodies to GTKO pig antigens suggested additional major barriers (i.e., anti-non-Gal antibodies), which would hinder progress towards clinical application. Nevertheless, this remains a major breakthrough as the identification of Gal and production of GTKO pigs demonstrated the potential of reducing porcine antigenicity through genetic modification.

The initial production of GTKO animals was performed through a tedious process of homologous recombination; however, recent advances in gene editing have dramatically sped the pace of xenotransplantation research (Table 1) [9, 11–13] setting the stage for highly efficient and rapid porcine genetic modification. Recently, the role of genetically engineered pigs has been reviewed, and this role effectively negates the human anti-pig humoral response to the threshold where hyperacute rejection and AHXR are no longer expected [9, 12–14]. In this climate of reduced *humoral* xenoantigenicity, an appraisal of pharmacologic strategies that will modulate the human *cell-mediated* response to porcine xenografts is increasingly relevant.

The cell-mediated response in allotransplantation is addressed with an effective pharmacologic armamentarium, mainly with calcineurin or mTOR inhibitors [15, 16]. Today, one of the most active frontiers in immunology and transplantation research is T cell costimulation signal modification. Much work over the past decade has defined costimulation signals, which regulate T cell activation and immune tolerance [17]. Although most of these agents are still experimental and early in the development pathway, preclinical studies utilizing experimental costimulation blockade agents have demonstrated prolonged engraftment of both solid organ and islet xenografts [18–25]. The approval of LEA29Y (belatacept) as a CTLA4-Ig protein for use in renal allotransplantation brought costimulation blockade to the clinic in the early 2000s [26, 27]. This was made possible after promising results from belatacept administration in preclinical nonhuman primate studies [28, 29].

In the last decade, researchers have increasingly utilized pig-to-nonhuman primate xenotransplantation models to study novel xenograft modification and novel costimulatory immunosuppression strategies in parallel. As discussions of pig-to-human xenotransplantation trials are underway [30, 31], we herein provide an overview of costimulation pathways, the current standing of clinical and preclinical development of these agents, and the preclinical data regarding their use in xenotransplantation.

## 2. T Cell Regulation through Costimulation Pathways

The adaptive immune system generates targeted responses first through (i) T cells identifying the antigen of interest and (ii) supplementary stimuli in the form of costimulation to induce antigen-specific T cell proliferation. Without these adjunct signals, T cells become anergic or undergo apoptosis and thus the response against that antigen is abrogated [32]. In this way, costimulation pathways support the role of T cell receptors (TCRs)—major histocompatibility complex (MHC) interaction by providing T cell the context of the antigen. Secondary and tertiary signals driven by cell surface costimulation molecules and soluble cytokines, respectively, determine the parameters of T cell activation [33]. Cytokines produced by the antigen-presenting cell (APC) and the T cell itself further propagate this activation cascade to induce a robust T cell response. Conventional immunosuppression works to abrogate the TCR and cytokine-induced signaling pathways preventing T cell activation [15, 16]. However, their lack of specificity to T cell mechanisms has led to well-recognized adverse side effects.

**Figure 1:**
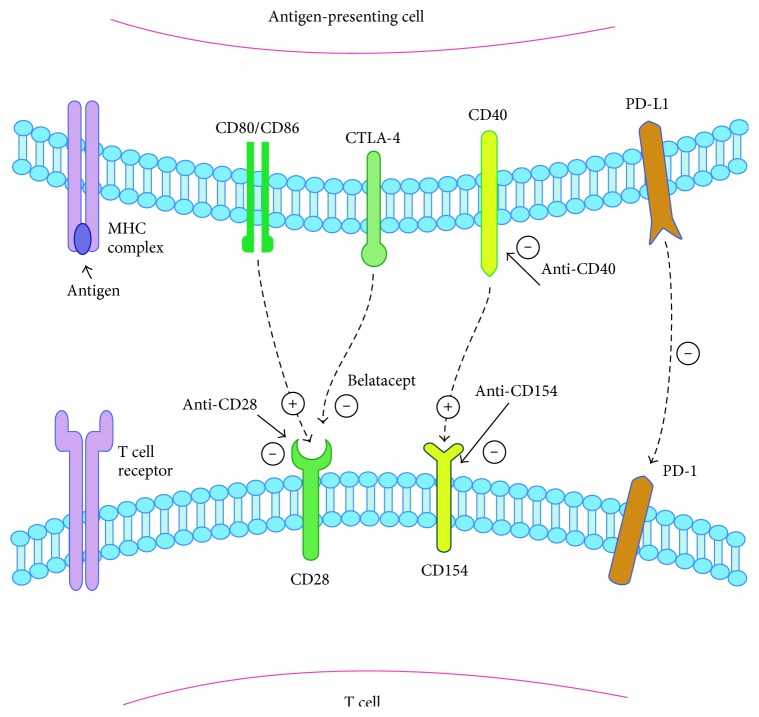
Costimulation pathways in T cell regulation. Upon MHC-antigen interaction with the TCR, costimulation pathways can augment or suppress the activation of the T cell. From left to right, CD28 is activated by CD80/CD86; however, after T cell activation, CTLA-4 is upregulated and with higher affinity than CD80/CD86 and binds to CD28 inhibiting the signal. CTLA-4Ig and belatacept work by taking advantage of their higher affinity to CD28 over CD80/CD86 and thereby block CD80/CD86 activation of CD28. CD154 and CD40 are other potent activators of T cells; monoclonal antibodies against either of these surface proteins have potential for application in transplant immunosuppression. PD-1 is expressed on T cells, and interaction with PD-1 Ligand (PD-L1) produces a suppressive signal to the T cell.

Costimulation pathways for T cell activation occur through a unique subset of cell surface markers, which are highly specific for the immune system and thus provide a target for immune modulators.  Figure 1 depicts the most commonly studied costimulation signals for potential use in transplant applications. The interaction of CD28 with CD80/CD86 has been the best defined. CD28 is highly expressed on naïve T cells. During TCR engagement with an APC, binding of CD28 to CD80/CD86 results activation and proliferation of the T cell. A feedback mechanism occurs at this juncture by which CD28 is then downregulated and the T cell increases expression of CTLA4-Ig. This molecule binds CD80/CD86 with much higher affinity than CD28 and produces an inhibitory signal as a highly evolved feedback mechanism [34].

Another increasingly significant costimulation pathway is the CD40/CD154 (CD40 ligand) interaction, which has been shown to be a potent stimulator of T and B cell activation through conventional APC interactions and also through interactions with innate immune cells and endothelium [35–38]. The inducible T cell costimulator (ICOS) molecule (CD278) has more recently been discovered to play an important role in T cell activation and differentiation as well as T and B cell interactions [39].

These costimulation pathways play a significant role during antigen recognition and T cell activation. Activated T cells rely on a specialized repertoire of surface proteins that assist in migration, adhesion, and interactions across the immunologic synapse to facilitate their effector function [40]. Lymphocyte function-associated antigen 1 (LFA1) is a well-studied molecule known to assist in immune cell endothelial attachment and migration and is recognized to play an important role in the stabilization of the immunologic synapse during antigen recognition and effector function (Figure 1) [41–43]. CD2 is more constitutively expressed on memory T cells, and interaction with LFA-3 is thought to not only have migration functions but also act as an activator of the potent memory T cell proliferation and response [40].

## 3. T Cell Costimulation in Organ Allotransplantation

Costimulation blockade has been extensively studied in preclinical allotransplantation models [41, 44–50]. Their relevance to xenotransplantation and xenoimmunity requires a thorough understanding of the salient findings from this growing body of research. One of the initial costimulation blockade agents was CTLA4-Ig, a protein that binds CD80/CD86 thus preventing CD28 costimulation and T cell activation. Preclinical data for CD40/CD154 blockade using anti-CD154 mAb also emerged in parallel with promising results. For example, an earlier study utilizing CTLA4-Ig and an anti-CD154 mAb (5C8 molecule) demonstrated synergistic prolongation of allograft survival in a nonhuman primate model, which continued even after withdrawal of immunosuppression [44]. Blockade of CD40/CD154 signaling pathway also was able to prolong graft survival in both renal and islet allotransplantation in nonhuman primates [44, 46, 51]. In these studies, the combination of both CTLA4-Ig and CD40 blockade appeared to prevent donor-specific antibody formation.

Memory T cells have been implicated in belatacept-resistant rejection; therefore, adjuvant therapy targeting memory T cell-specific features has been studied [40, 52]. An initial study of the LFA-3Ig molecule (alefacept) *in vitro* demonstrated suppression of alloreactive memory T cells, which were not suppressed by belatacept alone [45, 53]. Studies in nonhuman primates, however, demonstrated minimal benefit with an increased incidence of infectious complications [45, 47, 48, 53]. Based on early data, clinical use of the LFA-1 inhibitor, efalizumab, demonstrated some benefit in islet transplantation based on early data [42]. The use of LFA-1 inhibitor in combination with costimulation blockade also appeared to further prolong graft survival in islet allotransplantation [54]. LFA-1 exists in two forms: a commonly expressed, low-affinity form and a transient, high-affinity form, expressed only during activation. A recent study examined the use of more specific LFA-1 inhibitors (leukotoxin A and AL-579); targeting the high-affinity form of LFA-1 also did not demonstrate additional benefit in a renal transplant model [43]. Despite these data and the clinical potential, both alefacept and efalizumab were removed from the market by their manufacturers precluding further clinical study. A study using ICOS blockade with belatacept did not demonstrate any visible benefit to the combination of the two [50].

Costimulation blockade in clinical transplantation was first successfully introduced with the use of belatacept, a CTLA4-Ig molecule with higher affinity for B7 [26]. The initial BENEFIT trials demonstrated similar efficacy of belatacept-based regimens versus calcineurin inhibitors with an improved side effect profile [55–58]. However, a higher number of patients experienced an early severe rejection, which led to hesitation by many clinicians for widespread use [59]. Most of these rejection episodes were medically reversible which led to similar graft survival rates. The sparing of renal function demonstrated a potential benefit in long-term graft survival. Interestingly, patients who were on belatacept therapy also lacked significant production of donor-specific antibodies [29]. Further investigation into belatacept-resistant rejection demonstrated specific subsets of memory T cells that were present in patients who were not responsive to belatacept [40, 52, 60–62]. Alternative regimens incorporating belatacept in addition to conventional agents have shown promise [63–65], and further study to risk stratify these patients to individualize and introduce adjuvant therapy is ongoing.

Phase I clinical trials of a CD154 inhibitor demonstrated increased thrombotic phenomena not identified in preclinical testing and thus prevented clinical approval [66, 67] (as was subsequently demonstrated in xenotransplantation [68]). As preclinical data in allotransplant models appeared promising, newer agents to inhibit the CD40/CD154 and CD28/CD80/CD86 interaction and other costimulatory pathways are in the pipeline [69–72] but will need to complete their drug development cycle prior to consideration for human xenotransplant trials.

## 4. Costimulation Blockade in Xenotransplantation

The past two decades have been marked by great advances in the field of xenotransplantation with unprecedented graft survival times seen in preclinical models [1, 5, 13]. Tables 2, 3, and 4 summarize selected studies in solid organ (heart, kidney, and liver) and islet xenotransplantation with a specific use of anti-CD154mAb (Table 2), anti-CD40mAb (Table 3), or CTLA4-Ig (Table 4) between 2000 (the first use of costimulation blockade in xenotransplantation) to 2017. Continued development and improvement upon immunosuppressive regimens and the introduction of novel experimental agents appear to have contributed to this progress. Studies from the early part of the previous decade showed that induction therapy followed by high-dose conventional combination maintenance regimens was generally (but not uniformly) sufficient to sustain life-supporting pig grafts in nonhuman primates [73]. Conventional immunosuppressive therapy included agents such as cyclophosphamide, cyclosporine, mycophenolate mofetil, methylprednisone, and prednisolone (Tables 2, 3, and 4).

In 2000, Buhler et al. introduced the concept of costimulation blockade to the field of xenotransplantation [74]. Using a murine anti-human CD154mAb, they attempted to induce immune tolerance in nonhuman primates to transplanted pig peripheral blood mononuclear cells (PBMCs). More preclinical studies followed in both solid organ and islet xenotransplantation (Table 2) and increased markedly in the following decades. The most studied costimulatory modifiers within xenotransplantation have included anti-CD154mAb (Table 2), anti-CD40mAb (Table 3), and the CD28/B7 pathway (including CTLA4-Ig proteins abatacept and belatacept, as well as anti-CD28mAb, Table 4). Anti-CD154mAb therapy significantly prolongated porcine renal xenograft survival in nonhuman primates, with recent data demonstrating survival up to 405 days [22, 75, 76]. Unfortunately, this therapy is unlikely to be available for clinical xenotransplantation trials in the near future due to the agent's known thrombogenic properties [66–68]. High avidity CTLA4-Ig (belatacept) through interrupting the CD28/B7 pathway may be insufficient as monotherapy for xenograft maintenance [77]. Anti CD40mAb-based regimens have contributed to some of the longest reported xenograft survivals of pig heart and livers [24, 78]. Adhesion blockade with LFA-1 has also been utilized in a model of xenogenic islet transplantation, but with minimal benefit [79]. Further study continues in preclinical models to identify the most effective combination of costimulation blockade for xenotransplantation.

## 5. Costimulation Blockade and Genetic Modification of the Pig

Moving in parallel with this growing interest in xenotransplant costimulatory modification, *genome*-editing strategies aimed at costimulation pathways has also gained momentum. Xenotransplantation offers the unique potential to incorporate modifiers of the host immune response within the graft expression profile itself. To date, genetically modified pigs have been produced that alter the expression of endogenous porcine CTLA-4-Ig [80], or LEA29Y [81], or express human CD39 [82], or a human dominant-negative mutant class II transactivator [83]. Exhibiting variable successes, these approaches incorporate inhibitory regulation of the host costimulation interactions within the graft itself with the goal of facilitating suppression of host immune tolerance to the xenograft with less pharmacologic intervention than is required for allografts.

Regarding islet xenotransplantation, to date, five independent groups have reported survival of pig islets (genetically engineered or wild-type) for more than 3 months after transplantation into the liver of a nonhuman primate [19, 84]. Four groups utilized anti-CD154mAb-based immunosuppressive therapy (Table 2). Due to the likely unavailability of this agent, the Emory group has tried novel strategies with other clinically applicable or potentially clinically applicable medications such as basiliximab (anti-CD25mAb), LFA-1 blockade, and anti-CD40mAb (Table 3), in combination with belatacept.

Although several of these genetic strategies have provided promising results, the majority of gene-modification models are aimed at xenoantigen removal, complement regulation, or thromboregulatory properties of the xenograft. Indeed, these advances in genome-editing techniques have catalyzed a recent influx of novel and unique genetic backgrounds to the field of xenotransplantation. This rapid development raises a significant experimental issue; both novel genomic strategies and experimental immunosuppression strategies warrant individual appraisals. In the absence of a unified approach to gene modification within xenotransplantation, a cohesive appraisal of costimulatory intervention is challenging. The heterogeneity of genetic background thus prevents an effective stratification of costimulation blockade strategies for xenotransplantation. At present, a combination of graft modifications and exogenous immunosuppressive therapy to the host will be necessary to promote clinical application of xenotransplantation [1, 3, 13, 84, 85]. A standardized approach to testing genetic modification in combination with novel immunosuppressive agents will ideally bring clarity to the optimal combinations.

## 6. Conclusions

Currently published preclinical data demonstrate that immunosuppressive therapy, typically incorporating costimulation blockade agents, is required for successful engraftment of porcine tissues, even those with considerable genetic modification [9]. This convergence of experimental therapies in the preclinical setting presents a predicament when considering clinical xenotransplantation trials [31]. It is as yet uncertain whether conventional immunosuppressive agents may be effective enough to facilitate engraftment and maintenance of genetically modified (“humanized”) porcine organs or tissues. Furthermore, many of the immunosuppressive agents currently being tested in nonhuman primate models are not yet approved for clinical use. More rigorous testing of novel genetically modified pigs with minimal and/or more clinically relevant immunosuppression is warranted. However, the potential of costimulation blockade in xenotransplantation holds great promise for future use. Although genome-edited pig xenografts will certainly minimize the need for novel immunosuppressive agents, the increasing depth of our costimulation blockade library will benefit the future of allotransplantation and xenotransplantation alike.

## Figures and Tables

**Table 1:** Timeline for application of evolving techniques for genetic engineering of pigs employed in xenotransplantation.

Year	Technique
1992	Microinjection of randomly integrating transgenes
2000	Somatic cell nuclear transfer (SCNT)
2002	Homologous recombination
2011	Zinc finger nucleases (ZFNs)
2013	Transcription activator-like effector nucleases (TALENs)
2014	CRISPR/Cas9

CRISPR/Cas9: clustered randomly interspaced short palindromic repeats and the associated protein 9 (table adopted from Cooper et al.) [9].

**Table 2:** Selected studies using anti-CD154mAb in pig-to-nonhuman primate xenotransplantation.

First author (year)	Donor pig	Recipient NHP	Immunosuppressive regimen	Longest survival (days)
*Heart xenotransplantation, heterotopic*				
Buhler (2000) [86]	WT	Baboon	TBI, TI, splenectomy, IA, ATG, CVF, CSA, or anti-CD154mAb, MMF +/− pig stem cells	N.A
Houser (2004) [87]	CD55	Baboon	ATG, anti-CD2mAb, TI, CVF, anti-CD154mAb, MMF, CS	139
Dor (2005) [88]	GTKO	Baboon	ATG, anti-CD154mAb, MMF, CS	179
Kuwaki (2005) [89]	GTKO	Baboon	ATG, anti-CD2mAb, TI, CVF, anti-CD154mAb	179
Wu (2005) [90]	CD46	Baboon	ATG, anti-CD154mAb, +/− anti-CD20mAb +/− CTLA4-Fc	11
Wu (2007) [91]	CD46	Baboon	ATG, anti-CD154mAb, GAS194 or TPC, +/− IA	36
Ezzelarab (2009) [92]	GTKO	Baboon	ATG, CVF, anti-CD154mAb, MMF, CS	56
Mohiuddin (2012) [93]	GTKO.CD46	Baboon	ATG, anti-CD20mAb, anti-CD154mAb, MMF, CS	236
Kim (2013) [94]	GTKO	Cynomolgus	ATG, anti-CD20mAb, anti-CD154mAb, tacrolimus, CS	24
Ezzelarab (2015) [95]	GTKO	Baboon	ATG, anti-CD154mAb, MMF	56
Iwase (2015) [96]	GTKO.CD46.TBM	Baboon	ATG, anti-CD20mAb, anti-CD154mAb, MMF, CS	52
*Kidney xenotransplantation*				
Buhler (2000) [86]	WT	Baboon	TBI, TI, splenectomy, IA, ATG, CVF, CSA, or anti-CD154mAb, MMF +/− pig stem cells	N.A
Buhler (2001) [97]	CD55	Baboon	TBI, TI, splenectomy, IA, ATG, CVF, anti-CD154mAb, MMF, CS	29
Barth (2003) [98]	CD55	Baboon	Thymokidneys, anti-CD2mAb, ATG, anti-CD154mAb, CyP, CVF, MMF, CS	229
Gollackner (2003) [99]	CD55	Baboon	TI, splenectomy, IA, ATG, anti-CD154mAb, CyP, CVF, MMF, CS	13
Knosalla (2003) [100]	CD55	Baboon	TI, splenectomy, IA, ATG, anti-CD154mAb, CyP, CVF, MMF, CS	29
Yamada (2005) [75]	GTKO	Baboon	Vascularized thymic lobe, WBI, anti-CD2mAb, anti-CD154mAb, MMF, CS, CVF	68
Shimizu (2005) [101]	CD55	Baboon	Thymokidneys, splenectomy, IA, anti-CD3mAb, ATG, anti-CD154mAb, CyP, CVF, MMF	30
Griesemer (2009) [102]	GTKO	Baboon	Thymectomy, splenectomy, TBI, ATG, anti-CD2mAb, anti-CD154mAb, tacrolimus, MMF, anti-CD20mAb	83
Lin (2010) [103]	GTKO.CD46	Baboon	ATG, antiCD154mAb, MMF, CVF, CS	16
Nishimura (2011) [104]	GTKO	Baboon	Thymokidney, thymectomy, splenectomy, anti-CD3, antiCD2mAb, ATG, anti-CD20mAb, tacrolimus, MMF, anti-CD154mAb	15
Ezzelarab (2015) [95]	GTKO	Baboon	ATG, anti-CD154mAb, MMF	10
Higginbotham (2015) [22]	GTKO.CD55	Rhesus	Anti-CD4, anti-CD8, anti-CD154mAb, MMF, CS	310
Kim (2017) [76]	GTKO.CD55	Rhesus	Anti-CD4, anti-CD8, anti-CD154mAb, MMF, CS	405
*Liver xenotransplantation*				
Kim (2002) [105]	GTKO	Baboon	ATG, LoCD2b, CVF, anti-CD154mAb, azathioprine, tacrolimus, CS	9
Navarro-Alvarez (2016) [106]	GTKO	Baboon	ATG, LoCD2b, CVF, anti-CD154mAb, tacrolimus, CS	6
*Islet xenotransplantation*				
Buhler (2002) [18]	WT	Baboon	Splenectomy, IA, TBI, ATG, CVF, anti-CD154mAb, CSA, MMF, CS	28
Hering (2006) [107]	WT	Cynomolgus	Anti-CD25mAb, FTY720, rapamycin, anti-CD154mAb	187
Cardona (2006) [108]	WT	Rhesus	Anti-CD25mAb, anti-CD154mAb, CTLA4-Ig	>260
Rood (2007) [109]	GTKO	Cynomolgus	ATG, CVF, anti-CD154mAb, MMF, tacrolimus	>58
Casu (2008) [110]	WT	Cynomolgus	ATG, anti-CD154mAb, MMF	>60
van der Windt (2009) [19]	CD46	Cynomolgus	ATG, anti-CD154mAb, MMF	396
Thompson (2011) [20]	GTKO	Rhesus	Anti-CD154mAb, anti-LFA1mAb, MMF, belatacept	249
Bottino (2014) [111]	GTKO.CD46. TFPI.CTLA4Ig.CD39	Cynomolgus	ATG, MMF, anti-CD154mAb, CS	365
Shin (2015) [112]	WT	Rhesus	Anti-CD154mAb, ATG, rapamycin, CVF, adalimumab	>603

ATG: antithymocyte globulin; CS: corticosteroids; CSA: cyclosporine A; CVF: cobra venom factor; CyP: cyclophosphamide; NHP: nonhuman primate; TBI: total body irradiation; TI: thymus irradiation; mAb: monoclonal antibody; MMF: mycophenolate mofetil; mAb: monoclonal antibody; GTKO: *α*1,3-galactosyltransferase gene knockout; GAS914: a soluble glycoconjugate comprising Gal on poly-L-lysine backbone; N.A: not applicable; TBM: thrombomodulin; TPC: an aGal-polyethylene glycol polymer conjugate; WT: wild-type.

**Table 3:** Selected studies using anti-CD40mAb in pig-to-nonhuman primate xenotransplantation.

First author (year)	Donor pig	Recipient NHP	Immunosuppressive regimen	Longest survival (days)
*Heart xenotransplantation, heterotopic*				
Iwase (2015) [96]	GTKO.CD46.TBM	Baboon	ATG, belatacept, anti-CD40mAb, tacrolimus, MMF, CS	130
Mohiuddin (2016) [78]	GTKO.CD46.TBM	Baboon	ATG, anti-CD20mAb, anti-CD40mAb, CS	>900
*Kidney xenotransplantation*				
Iwase (2015) [23]	GTKO.CD46.CD55 TBM.EPCR.CD39	Baboon	ATG, anti-CD20mAb, anti-CD40mAb, rapamycin, tocilizumab, etanercept	136
*Liver xenotransplantation*				
Shah (2017) [24]	GTKO	Baboon	ATG, anti-CD40mAb, tacrolimus, CVF, CS	29
*Islet xenotransplantation*				
Thompson (2011) [21]	WT	Rhesus	Anti-CD25mAb, anti-CD40mAb, rapamycin, belatacept	203

NHP: nonhuman primate; WT: wild-type; ATG: antithymocyte globulin; CVF: cobra venom factor; MMF: mycophenolate mofetil; mAb: monoclonal antibody; CS: corticosteroids; GTKO: *α*1,3-galactosyltransferase gene knockout; TBM: thrombomodulin; EPCR: endothelial cell protein C receptor.

**Table 4:** Selected studies using CTLA4-Ig in pig-to-nonhuman primate xenotransplantation.

First author (year)	Donor pig	Recipient NHP	Immunosuppressive regimen	Longest survival (days)
*Heart xenotransplantation, heterotopic*				
Iwase (2015) [96]	GTKO.CD46.CD55	Baboon	ATG, anti-CD20mAb, abatacept, MMF, CS	23
Iwase (2015) [96]	GTKO.CD46.TBM	Baboon	ATG, belatacept, anti-CD40mAb, tacrolimus, MMF, CS	130
*Liver xenotransplantation*				
Shah (2017) [24]	GTKO	Baboon	ATG, belatacept, tacrolimus, CVF, CS	25
*Islet xenotransplantation*				
Cordona (2006) [108]	WT	Rhesus	Anti-CD25mAb, anti-CD154mAb, CTLA4-Ig	>260
Hecht (2009) [113]	Fetal pancreatic fragments	Cynomolgus	Anti-CD25mAb, anti-CD154mAb, FTY720, rapamycin, CTLA4-Ig	380
Thompson (2011) [21]	WT	Rhesus	Anti-CD25mAb, anti-CD40mAb, rapamycin, belatacept	203
Thompson (2011) [20]	GTKO	Rhesus	Anti-CD154mAb, anti-LFA1mAb, MMF, belatacept	249
Thompson (2012) [79]	WT	Rhesus	MMF, belatacept, alefacept, anti-LFA1mAb, tacrolimus	114
Graham (2013) [114]	WT	Cynomolgus	Anti-CD25mAb, abatacept, tacrolimus, rapamycin	>180

NHP: nonhuman primate; WT: wild-type; ATG: anti-thymocyte globulin; CVF: cobra venom factor; MMF: mycophenolate mofetil; mAb: monoclonal antibody; CS: corticosteroids; GTKO: *α*1,3-galactosyltransferase gene knockout; TBM: thrombomodulin.

## Abbreviations

APC: Antigen-presenting cells
AHXR: Acute humoral xenograft rejection
Gal: Gal *α*(1,3) Gal
GTKO: *α*1,3-Galactosyltransferase gene knockout
LFA: Lymphocyte function-associated antigen
mAb: Monoclonal antibody
MHC: Major histocompatibility complex.
