# Corrigendum to “The Role of Costimulation Blockade in Solid Organ and Islet Xenotransplantation”

**DOI:** 10.1155/2018/6343608

**Published:** 2018-03-22

**Authors:** Kannan P. Samy, James R. Butler, Ping Li, David K. C. Cooper, Burcin Ekser

**Affiliations:** ^1^Division of Transplant Surgery, Department of Surgery, Indiana University School of Medicine, Indianapolis, IN, USA; ^2^Xenotransplantation Program, Department of Surgery, The University of Alabama at Birmingham, Birmingham, AL, USA

In the article titled “The Role of Costimulation Blockade in Solid Organ and Islet Xenotransplantation” [1], there was an error in Figure 1 and its legend. The corrected figure is shown below.

## Figures and Tables

**Figure 1:**
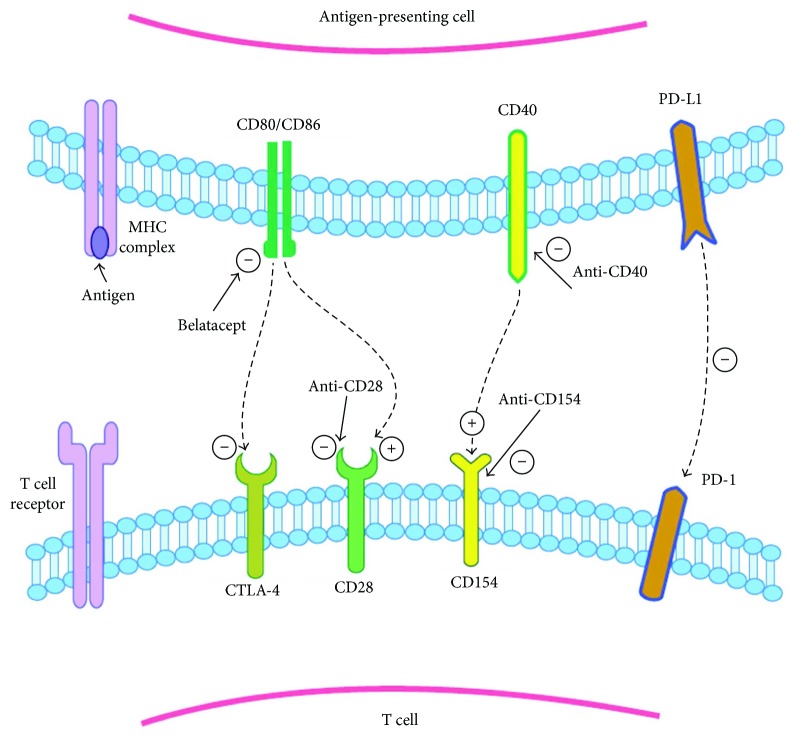
Costimulation pathways in T cell regulation. Upon MHC-antigen interaction with the TCR, costimulation pathways can augment or suppress the activation of the T cell. From left to right, CD28 is activated by CD80/CD86. CTLA-4 coinhibitor competes with CD28 for binding to CD80/CD86. CTLA-4Ig and belatacept work by taking advantage of their higher affinity to CD28 over CD80/CD86 and thereby block CD80/CD86 activation of CD28. CD154 and CD40 are other potent activators of T cells; monoclonal antibodies against either of these surface proteins have potential for application in transplant immunosuppression. PD-1 is expressed on T cells, and interaction with PD-1 ligand (PD-L1) produces a suppressive signal to the T cell.
